# Off-gassing plasticware to decrease the toxicity effect in embryo culture

**DOI:** 10.5935/1518-0557.20210005

**Published:** 2021

**Authors:** Jhon Troya Santos, Lisa Soobrian, Sonya Kashyap

**Affiliations:** 1 Genesis Fertility Centre, Vancouver, British Columbia, Canada

**Keywords:** off-gassing, plasticware, mouse embryo assay, human sperm survival assay

## Abstract

**Objective:**

To determine if reproductive toxicity might be reduced by off-gassing plasticware before use in in vitro fertilization culture.

**Methods:**

Petri dishes were grouped according to the off-gassing days before use for testing as follow: 0 day (Group A); 3 days (Group B); 7 days (Group C). Two bioassays were run: Human survival sperm assay (HSSA) at 24 hours for all groups and mouse embryo assay (MEA) to evaluate the effect of the off-gassing procedure. Sperm motility and sperm motility index values were examined and blastocyst formation was calculated on Days 4 and 5.

**Results:**

HSSA revealed decreased sperm motility in Group A (84%) compared to Groups B (94%) and C (93%) (*p*<0.5). MEA showed no statistical difference in blastocyst formation for the off-gassing groups (79.6%, 84.4%, 80%, Group A, B, and C, respectively; *p*>0.5). Hatching blastocyst formation on Day 5 was decreased in the non-off-gassing group (49% vs. 56.7%, *p*>0.5).

**Conclusions:**

Off-gassing for at least 72 hours decreases the toxicity of plasticware before use in in vitro fertilization cultures for HSSA. Further investigation needs to be done in order to standardize the bioassays used to evaluate this procedure.

## INTRODUCTION

The successful culture of human embryos and sustained pregnancy rates in an in vitro fertilization (IVF) program rely on different direct and indirect factors that include ovarian stimulation, patient age, spermatozoa, and the conditions to which they are exposed as they grow and develop from gametes to embryos. The latter include biochemical and biophysical variables in the IVF laboratory.

Several volatile organic compounds (VOCs) are known to affect embryo development (Mortimer *et al.*, 2018). High VOC levels in the air of the laboratory and inside the incubator might impact embryo quality and outcome (Cohen *et al.,* 1997; Hall *et al.,* 1998). Removal of VOCs might help to improve outcomes (Heitmann *et al.,* 2015).

Plasticware is known to contain harmful chemicals and emit VOCs. Research has shown that plasticware in the laboratory may vary in quality between types of dishes, lots within a specific type of dish, and even within wells in a multi-well dish. Toxic chemicals found in plasticware may present reproductive toxicity and cause delays in embryo development. In order to provide the best culture conditions for IVF, Quality Control (QC) of products used in the IVF laboratory is routinely performed by manufactures and often by IVF laboratories with the aid of bioassays. Bioassays that may be used to test reproductive toxicity include the Human Sperm Survival Assay (HSSA) (Bavister & Andrews, 1988; Claassens *et al.*, 2000; Lierman *et al*., 2007; Davidson *et al*., 1988), the Mouse Embryo Assay (MEA) (Davidson *et al.,* 1988; Fleetham *et al*., 1993; Scott *et al*., 1993), and continued culturing of abnormally fertilized zygotes (Bertheussen *et al.,* 1989).

Studies have shown that off-gassing plasticware in a flow hood prior to embryo culture might reduce the potential reproductive toxicity effects from plasticware (Holyoak *et al.,* 1996), although results were inconclusive and no recommendation was made suggesting the use of off-gassing. We used HSSA and MEA to verify whether reproductive toxicity might be reduced by off-gassing plasticware, and to identify the optimal duration of off-gassing to eliminate reproductive toxicity from plasticware.

## MATERIAL AND METHODS

### Off -gassing plasticware

Embryo culture GPS dishes with the same batch number (8 x 50µl wells and 3 x 100µl wells; EGPS, Life Global, USA) were used in HSSA and MEA. They were opened in aseptic conditions on the hood and exposed to circulating air before off-gassing for 0 day (Group A), 3 days (Group B), and 7 days (Group C). All dishes were left at room temperature (23ºC) until sampling and testing. 

### Human Sperm Survival Test

Human sperm was obtained from 40 individuals who had donated sperm for research purposes. Twenty-two normal semen samples were included in the sperm survival test (sperm concentration of 15 x106/mL; 40% of the sperm was motile; and normal values for strict sperm morphology of 4%) (Tygerberg) [lower reference limits from the fifth centile value accepted by World Health Organization (WHO) 2010 Standards 5th Ed]. Semen profiles were defined within one hour of production following the 2010 WHO guidelines. Sperm samples were centrifuged at 300xg for 20 min using previously prepared dual gradient columns at 80% and 40% (Allgrad, AGSS-250, Life Global, USA) and a double wash step with supplemented HTF Media (HTF Life Global, MNHT-250 with 5.0 mg Life Global Protein Supplement); all reagents were incubated at 37ºC before use. Each sample suspension was resuspended and split into two culture medium tubes (quality control and off-gassing day group); volume was adjusted to a final concentration of 5 x106/mL and used according to the designed test.

For the quality control sample, 6.5mL of the resuspended sperm sample was added directly into a previously QC tested and released GPS culture dish (> 85% sperm motility survival post 24 hours). The sample was incubated for sampling at 0 and 24 hours at room temperature (23.3ºC) for motility analysis in a Mackler chamber. For the off-gassing group testing, 6.5mL of resuspended sperm sample also was added directly into each of the off-gassing group GPS dish. The sample was incubated for sampling at 24 hours at room temperature (23.3ºC) for motility analysis in a Mackler chamber.

The “Sperm motility index” (SMI) was calculated for the off-gassing groups. It was derived by dividing the progressive motility in the tested off-gassing group at the end of the test period by the progressive motility in the quality control sample at the start of the test period.

### Mouse Embryo Assay

Cryopreserved 1-cell hybrid mouse embryos (n=139) were obtained from Embryotech Laboratories (Haverhill, MA, USA). They were grouped in three different batches: Batch 1 (n=16) B1-1061708; Batch 2 (n=75) B1-1038219; and Batch 3 (n=48) B1-1071416. The embryos were thawed according to the manufacturer instructions and allowed to equilibrate for 10 min at room temperature in HTF with HEPES (HTF-HEPES; Life Global, USA) containing 0.1mg/mL polyvinyl alcohol (PVA) (Life Global Group, USA). After equilibration, the embryos were placed in individual wells in Global Total medium (LGGT, Life Global, USA) with oil covering the embryo GPS dish and moved immediately into a Minc mini incubator (Cook Medical Group).

Embryo cultures were prepared the day before thawing and were equilibrated overnight at 37ºC, 6.5% CO2 and 5% Oxygen. Pre-tests with a portable EPOC arterial blood gas analyzer (Siemens Healthcare GmbH, Zurich, Switzerland) were run to confirm a pH of 7.32 in the incubator to ensure a proper embryo culture environment. For each of the off-gassing groups, the individual wells in the GPS dish contained 35µl of Global Total medium covered with 1.2mL of mineral oil (LGUA, Life Global Lifeguard, Life Global, USA).

Blastocyst formation was calculated and compared between the off-gassing day groups on day 4 or 5 of embryo culture and according to embryo batch. The groups were also compared for hatching blastocysts.

### Statistical Analysis

All data analyses were performed using SPSS version 21.0 (IBM, New York, USA). Continuous data were tested for normality. Normally distributed data were presented as mean ± standard deviation (SD); non-parametric analysis was performed using the Kruskal-Wallis Test. Categorical variables were expressed as percent frequencies and analyzed with the chi-squared test. *p*-values<0.05 were considered to be of statistical significance.

## RESULTS

### Human sperm survival assay and off-gassing group

**Figure 1:**
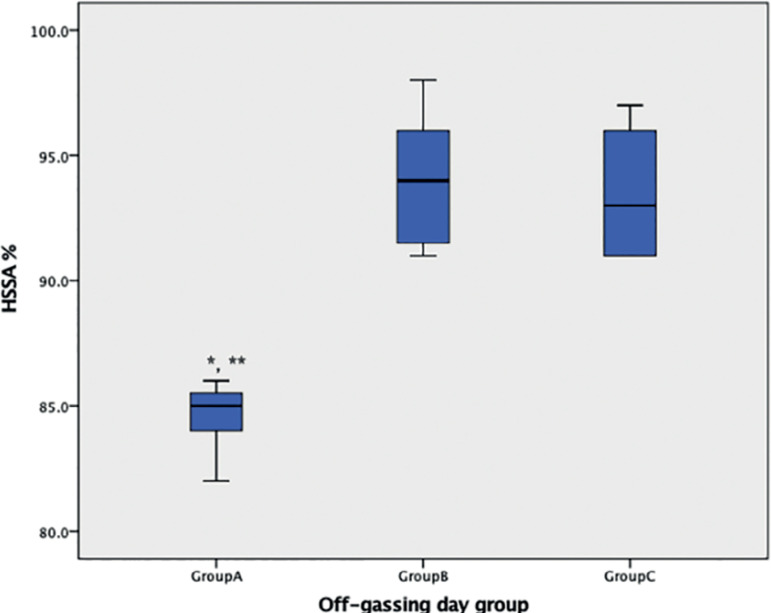
HSSA from the off-gassing groups. The value represents HSSA index multiple by 100%. Bonferroni multiple comparison. The mean difference is significant at the 0.05 level. *Group A and B significantly different. **Group A and C significantly different.

Mean motility was calculated and compared between the groups during the first 24 hours. Sperm motility index results are shown in Figure 1. Results showed that motility was not affected when sperm were cultured in dishes for more than three days of off-gassing. Groups B and C were not different (indices of 0.94 and 0.93, respectively). However, when sperm were exposed in dishes zero day after off gassing, the HSSA decreased and the motility index of Group A (0.84) was statistically different from the indices found in Groups B and C (*p=*0.015 and *p*=0.024, respectively).

### Mouse Human Assay

#### MEA on day 4 and off-gassing group

Overall blastocyst development rate on day 4 was 76.3%. No statistical differences were found between days of off-gassing in Groups A, B and C for blastocyst development rate (77.6%, 73.3%, and 77.8%, respectively; *p*=0.854, X2=0.315). Furthermore, when the embryos on day 4 were regrouped and compared against the group with zero day of off-gassing (Group A) and the groups with other off-gassing periods (Group B+C), results showed that the off-gassing procedure did not increase blastocyst formation on day 4 (75.6% and 77.6%, respectively; *p*=0.792, X2=0.07), as described in Tables 1 and 2.

**Table 1:** Demographics and cycle characteristics of the study population.

BLASTOCYST RATE	Off-gassing Day group			
	Group A	Group B	Group C	*p*-value
Blastocyst Formation rate on day 4 (%)	77.6	73.3	77.8	0.854
Blastocyst Formation rate on Day 5 (%)	79.6	84.4	80	0.804

The mean difference is significant at the 0.05 level.

**Table 2:** Blastocyst formation for regrouped 0 days off-gassing and any off-gassing on day 4 and day 5.

BLASTOCYST RATE	Off-gassing group		
	Any day	0 day	*p*-value
Blastocyst Formation rate on day 4 (%)	75.6	77.6	*0.792*
Blastocyst Formation rate on Day 5 (%)	82.2	79.6	*0.704*

The mean difference is significant at the 0.05 level.

#### MEA on day 5 and off-gassing group

Overall blastocyst development rate on day 5 was 81.3% for all groups. Blastocyst development rates in Group A were slightly lower than the rates seen in Groups B and C (79.6%, 84.4%, 80%, respectively), although the difference was not significant (*p*=0.804, X2=0.437). When the embryos on day 5 were regrouped and compared between zero day of off-gassing (Group A) and other periods of off-gassing (Group B+C), results showed that off-gassing did not increase blastocyst formation on day 5 (79.6% vs. 82.2%, *p*=0.704, X2=0.144), as described in Tables 1 and 2.

#### MEA on days 4 and 5 according to different mice batches

Blastocyst development rate on day 4 was different among the three mice batches. Mice from Batch 1 achieved higher blastocyst formation (100%) and when were compared results to Batch 2 (80%) and Batch 3 (62.5%) yielded a significant difference (*p*=0.005, X2=10.5). On day 5, when blastocyst formation was categorized as expanded or higher blastocyst, embryos in Batch 1 also reached 100% blastocyst formation, a proportion statistically higher when compared to Batches 2 and 3 (74.7% and 85.4%, respectively; *p*=0.041, X2=6.3), as described in Table 2. Different blastocyst formation rates were found in the mice batches, with increased blastocyst formation seen in Batch 1.

#### MEA on day 5 according to hatching formation

**Figure 2:**
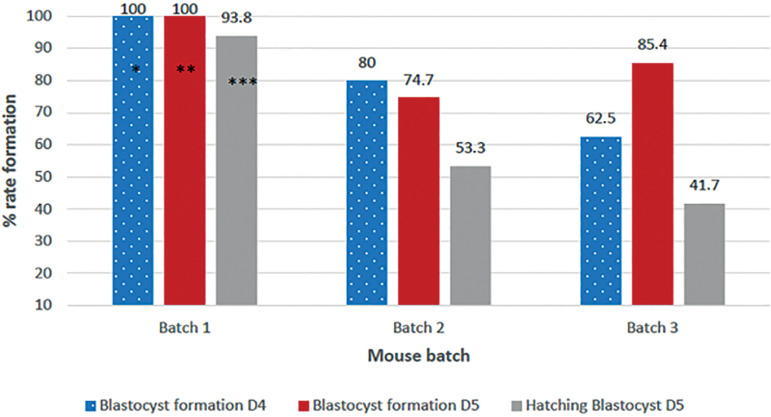
Embryo development formation according to the mouse batch on day 4 and day 5. *Day 4 blastocyst formation statistically significant among batch1,2,3 (*p*-value<0.05). **Day 5 blastocyst statistically significant among batch1,2,3 (*p*-value<0.05). ***Hatching rate statistically significant among batch1,2,3 (*p*-value<0.05).

**Figure 3:**
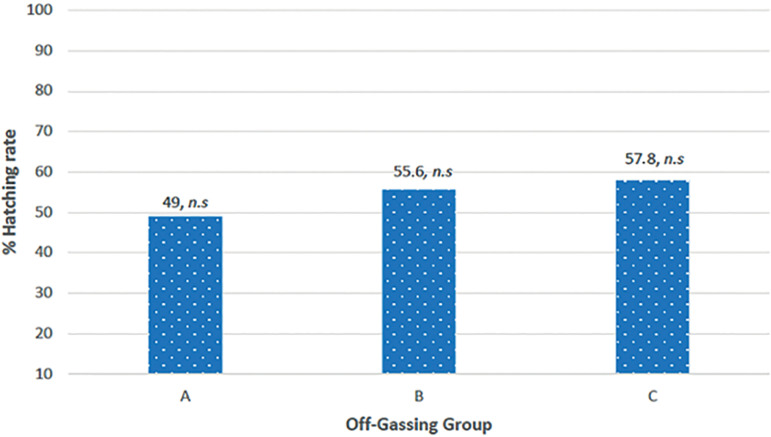
Hatching formation for the off-gassing groups. No statistical (n.s) difference at the 0.05 level.

In this study we assessed hatching formation as a new parameter to introduce in MEA. Our results showed improved hatching formation when the embryos were cultured in dishes submitted to off-gassing. The data revealed an overall hatching blastocyst development rate on day 5 of 54%. Hatching blastocyst development in Group A was slightly lower than in Groups B and C (49%, 55.6%, and 57.8%, respectively), but no significant differences were seen between the groups (*p*=0.671, X^2^=0.799), as seen in Figures 2 and 3. Furthermore, when embryos were regrouped and compared between zero day of off-gassing (Group A) and other periods of off-gassing (Group B+C), results showed higher hatching blastocyst formation in the off-gassing groups (56.7% and 49%, respectively), although differences were not significant (*p*=0.385, X^2^=0.755).

Hatching blastocyst rate also was calculated for each mice batch, and results also showed improved hatching formation in Batch 1 as seen on day 4 and 5; hatching formation was 93.8%, a proportion statistically higher than the ones seen in Batches 2 and 3 (53.3% and 41.7%, respectively; *p*=0.001, X^2^=13), as described in Table 2.

## DISCUSSION

Petri dish is a standard component in embryo culture systems used for decades in IVF laboratories. They are made from polystyrene, synthesized by using a reaction known as free radical polymerization. Suppliers have been using HSSA and MEA as acceptable criteria to assess plasticware toxicity. However, toxicity is poorly defined and might vary from batch to batch and over time despite prior approvals for use in IVF.

HSSA remains as one of the preferred QC methods used routinely in fertility laboratories, manufactures, and externally administered proficiency testing programs (CP) despite its limitations. The acceptable cut-off point is arbitrary. Items are considered to be predictive of reproductive toxicity and banned for use when the index decreases to less than 85%. Despite careless standard agreement, this criteria was defined by the work of Critchlow *et al*. (1989) as well. Therefore, some IVF plasticware suppliers use lower acceptance criteria such as 70% (Thermo scientific), while other testing and quality control laboratories use 60% instead (Embryotech laboratory, MA, USA) (Tech note Number 60).

It is important to mention that MEA is currently regarded as the most adequate assay used to exclude toxic items. It is a requirement for products approved by the Food and Drug Administration (FDA) for clinical use in IVF. In fact, the FDA recently issued a draft guideline with some recommendations on conducting MEA for supporting devices used in IVF (FDA, 2019).

Unfortunately, there is still no consensus about the procedure. For example, most laboratories and manufactures use different cutoff acceptance criterion: 70%, 75% or 80% blastocyst formation, none based on statistical criteria (Ainsworth et al., 2017; Fleetham et al., 1993; Sathananthan et al., 2003). In the scientific community there is strong disagreement about the use of MEA results to predict human embryos, although several strategies have been reviewed to improve the sensitivity of MEA (Wolff et al., 2013). Concerns exist with sensitivity and the lack of standardization, which involves subjective analysis of morphology (Esfandiari & Gubista, 2020). Our results showed that MEA was affected by the used mouse batch, which is also mentioned in other reports, where outbred embryos were more sensitive to toxins than either inbred or hybrid mouse embryos (Dandekar & Glass, 1987; Khan et al., 2013).

It has been mentioned that it is not apparent all the time and it is difficult to isolate, in some occasions mouse embryo development could be impaired, with slower rate of development or reduced number of cells within the blastocyst; impaired human embryos also have been seen (Wiemer, 2017).

In this study, we introduced a new indicator to be considered for MEA, as we evaluated the capacity of the blastocyst on day 5 to hatch. The premise of a reduced potential embryo due to toxicity might be explained by hatching deficiency; the structure and physiology of the whole blastocyst seem to play a vital role on the hatching process. In fact, spontaneously hatching embryos have better pregnancy rates than expanded blastocyst (Chimote et al., 2013; Lee et al., 2013). In our results, even if we found an increased hatching blastocyst formation on day 5 for the embryos grown in off-gassing dishes, there were no significant differences. It is well known that differences in the results of the bioassay might be greatly affected by different factors such as: protein used in the media; the type or quantity of the toxic component needed to be detected etc. (Adams et al., 2007; Rollene et al., 2008; Gada et al., 2008; Mestres et al., 2019).

Off-gassing plasticware before use in embryo culture is a simple and affordable improvement of the routine work in IVF laboratory. However, it is not clearly defined its contribution to maintain a quality work. Nevertheless, this is the first report to indicate off-gassing plasticware as a standard procedure for IVF laboratories and to introduce the hatching rate as a new variable for MEA. Many laboratories and unpublished data have suggested off-gassing plasticware allows materials such as aldehydes, toluene, cyclenes, styrene, and other hydrocarbons to dissipate and have decreased presence in embryo culture (Gilligan et al., 1997; Khoudja et al., 2013).

This study found that allowing plasticware off-gassing prior to use might improve HSSA results. The results showed that sperm motility was maintained when the dishes were exposed to air without any differences even if they were exposed over the time.

Although our MEA results did not find blastocyst formation differences between the off-gassing groups when they were compared on Day 4 or Day 5, the overall blastocyst formation after Day 5 was not affected by off-gassing the dishes. The reported 81.3% blastocyst formation is still within the acceptable criteria not to be considered toxic, according to the reference value used by the majority of laboratories.

In conclusion, we found that off-gassing performed for at least 72 hours might reduce the toxicity effect of plasticware, and its effects can be tested by HSSA. Our study also suggests the addition of another endpoint to analyze MEA results and help to improve this bioassay instead of using only blastocyst formation. However, the results need to be analyzed and interpreted carefully. Further investigation needs to be done in order to standardize this bioassay. Although many laboratories use this procedure in their quality control efforts, this is the first report indicating the effect of off-gassing dishes on the performance of embryo culture.
